# Entanglement of CCR5 and Alzheimer’s Disease

**DOI:** 10.3389/fnagi.2019.00209

**Published:** 2019-08-07

**Authors:** Tianwen Li, Jianhong Zhu

**Affiliations:** ^1^Department of Neurosurgery, Huashan Hospital, Fudan University, Shanghai, China; ^2^State Key Laboratory of Medical Neurobiology, Shanghai, China; ^3^Shanghai Medical College, Fudan University, Shanghai, China; ^4^Institutes of Brain Science, Shanghai, China

**Keywords:** CCR5, Alzheimer’s disease, neuroinflammation, microglia, neurodegeneration

## Abstract

Although the mechanisms of Alzheimer’s disease are diverse and unclear, the past 20 years have witnessed the unprecedented development of the AD inflammation theory. As a key inflammatory receptor family, the C-C chemokine receptor family is a remarkable participant in the cause of Alzheimer’s disease; of this family, CCR5 is the most widely studied. CCR5 is an essential entrance when HIV infects immune cells and is also involved in other inflammatory and immune activities. New evidence on the inevitably intertwined link between Alzheimer’s disease and CCR5 indicates that CCR5 accelerates the development of Alzheimer’s disease, and few studies disputed it. The role of CCR5 in Alzheimer’s disease remains elusive. However, as the research progresses, this intricate relationship will gradually be uncovered.

## Introduction

C-C chemokine receptors 5 (CCR5) is a kind of cytokine belonging to the β chemokine receptor family of integral membrane proteins (Power et al., 1995). CCR5 is well known (or notorious) mainly because it collaborates with human immunodeficiency virus-1 (HIV-1) when the virus enters target immunological cells (Murphy, 2001; Ghalib, 2009). Intriguingly, most current anti-HIV drugs target CCR5. At the end of the 2018, the announcement of Chinese gene edited infants whose CCR5 genes were deleted by CRISPR-Cas9 again brought the debate about CCR5 to the forefront.

Alzheimer’s disease (AD), characterized by typical pathological changes such as senile plaques and neurofibrillary tangles, is the most prevalent neurodegenerative disorder. With the aging of the population, the battle with AD will become more difficult. Although the causes and pathogenetic mechanisms remain uncertain, there is growing evidence linking neuroinflammation with AD (Khoury, 2010; Heneka et al., 2015). Numerous cytokines, e.g., tumor necrosis factor (TNF) (Irene et al., 2015), interleukin-1 (IL-1) (Irene et al., 2015), interleukin-6 (IL-6) (Haddick et al., 2017), and colony-stimulating factor (CSF) (Vincent et al., 2009), have been suggested to be connected with the course of AD. Additionally, many variants in immune genes such as TREM2, CD33 and CR1 were recognized by genome-wide association studies (GWAS) as genetic risk factors for AD (Jean-Charles et al., 2009; McGuinness et al., 2009; Hollingworth et al., 2011; Guerreiro et al., 2013; Hickman Suzanne and Joseph, 2014).

Our receptor of interest, the chemokine receptor, is involved in substantial inflammatory diseases of the central nervous system (CNS) and functions in the recruitment and immigration of immune cells. These receptors are mainly distributed in microglia and recruited peripheral blood monocytes in the CNS (El, 2010), while they are rarely expressed on neuronal cell membranes under normal physiological conditions (Huerta et al., 2004). Increased expression of CCR5 in the CNS is an inflammatory response to many neuropathological diseases, e.g., stroke (Joy et al., 2019), Parkinson’s disease (Huerta et al., 2004), multiple sclerosis (SRensen et al., 1999; Trebst et al., 2001), and Rasmussen encephalitis (Bien Christian et al., 2013; Varadkar et al., 2014). At the beginning of this year, an article published in Cell (Joy et al., 2019) further attracted everyone’s attention to the association between CCR5 and neuronal plasticity that is potentially relevant to neuroinflammation and AD. In this review, we will elucidate the intricate association between AD, inflammation, and CCR5.

## AD and Neuroinflammation

### Pathogenesis of AD

As the most common cause of dementia, AD is still defined by the combined presence of amyloid and tau; thus, countless studies are conducted with the aim of discerning the enigma of these two pathological factors. However, all current drug clinical trials for amyloid or tau have ended in failure. Researchers are gradually moving away from the simple estimation of linear connection as proposed in the initial amyloid hypothesis (Small and Duff, 2008). Several lines of evidence confirmed that vascular damage increased the risk of later cognitive impairment and finally dementia (Exalto et al., 2013; Gottesman Rebecca et al., 2014; Kuhn et al., 2014; Rawlings Andreea et al., 2014). Additionally, there are considerable disease and lifestyle determinants that could result in increased susceptibility to developing AD, e.g., traumatic brain injury, hypertension, diabetes, obesity, education, exercise, and psychological factors, except for the proverbial aging and risk genetic allele (Norton et al., 2014). Notably, most of these factors are relevant to inflammation and immunity. Consequently, a variety of hypotheses and theories of AD have been proposed, among which the inflammation hypothesis is receiving growing support (Newcombe et al., 2018; Oliveira et al., 2018; Schultzberg et al., 2018; Spangenberg and Green, 2018). Although the reviews of neuroinflammation and AD are abundant and comprehensive (for more detail, we recommend Ref; Josef Karkos, 2003; Heppner Frank et al., 2015; Ransohoff, 2016; Cuello, 2017), we will focus on the relationship between microglia and neuroinflammation because microglia harbor the majority of CCR5 in the CNS.

### Microglia as a Double-Edged Sword

Notwithstanding existing evidence indicating that peripheral immune cells can infiltrate brain tissue through the damaged blood-brain barrier (BBB) under pathological conditions and cause inflammation (Villeda Saul et al., 2011), native microglia and activated astrocytes, which widely reside everywhere throughout the brain, are the main contributors to neuroinflammation. Unlike neurons, astrocytes, and oligodendrocytes, microglia are considered resident immune cells from the mesoderm and function in some housekeeping work, including neurogenesis, the trimming and stimulation of synapses, the modulation of cognitive processes, and immunological surveillance. Gradual deterioration of the immune system increases vulnerability to infections and diseases as individuals age. Some altered expression of microglia-related genes that could accelerate the progression of AD has been identified in AD patients (Cribbs et al., 2012). Microglia isolated from postmortem aging brains also showed significant transcriptome characteristics compared with those obtained from younger brains; genes associated with cell-adhesion axon-guided cell surface receptor expression and actin assembly were specifically affected (Galatro et al., 2017). Moreover, microglial cells from the aged cortex show many morphological abnormalities, including the formation of nodal processes and division processes of acellular globules (Streit Wolfgang et al., 2010).

**FIGURE 1:**
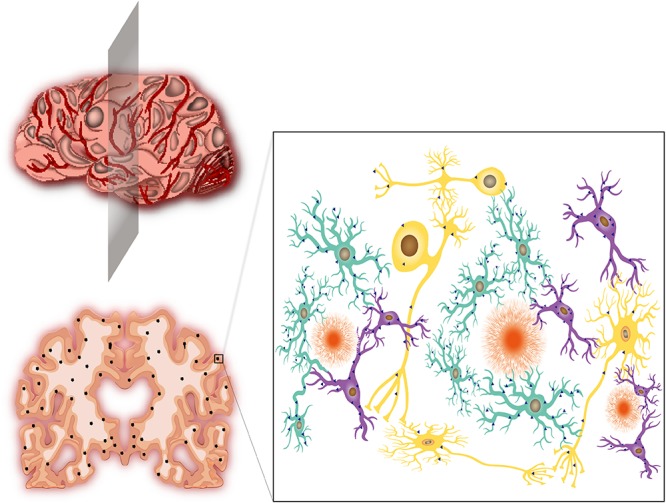
Alzheimer’s disease, neuroinflammation and CCR5. **(Upper left)** The schematic graph of an AD patient’s brain. The brain shows varying degrees of shrinkage and vascular inflammation. The gray patches represent neuroinflammation in different regions of AD brain insults. **(Lower left)** Brain slice of a severe AD patient from the location of shaded plane in the **(Upper left)** graph. Apparent shrinkage and numerous Aβ depositions are presented. The black dots represent Aβ deposits. **(Right)** The magnified version of the box in the lower left graph. The Aβ deposits are surrounded by activated astrocytes (agate green) and microglia (purple). The neurons (yellow) are inevitably affected by these neighbors, which leads to disorders of electrical and chemical signal conduction. The blue triangles represent the expression of CCR5. Activated astrocytes and microglia produce abundant CCR5, while neurons express few of them. The radial orange circles are Aβ deposits.

The proteins that microglia produce exhibit dramatic changes during aging, neurodegeneration, and neuroinflammation. For example, secreted cytokines, displayed membrane proteins, and energy and metabolism protein needs differ drastically (Ritzel et al., 2015; Mrdjen et al., 2018). However, the complex relationship between microglia, tau, and amyloid beta protein (Aβ) remains controversial. On the one hand, many studies have indicated that microglia are capable of congregating around Aβ and phagocytosing it to attenuate the pathological load. On the other hand, activated microglia could damage neurons and vascular epithelial cells, which lead to additional impairment of cognitive function (Grathwohl and Kalin, 2010; Seabrook et al., 2010; Dagher et al., 2015; Olmos-Alonso et al., 2016; Spangenberg et al., 2016). The distinct subtypes of microglia contribute to the AD course in a diverse manner, and the heterogeneity of pro- and anti-inflammatory timing of microglia is a formidable obstacle (Martin et al., 2010; Heneka Michael et al., 2013; Medeiros et al., 2013; Chakrabarty et al., 2015; Marie-Victoire et al., 2015; Fu et al., 2016). Reactive microglia can accelerate the propagation of tau pathology, thereby causing a deterioration in inflammation (Yoshiyama et al., 2007; Sanchez-Mejias et al., 2016). Deposition of Aβ worsens neurodegeneration and triggers pro-inflammatory responses. Moreover, Aβ binds to inflammatory receptors (Yan et al., 1996; Deane et al., 2003; Shirong et al., 2012) and promotes immune-related transcriptional signals (Bonaiuto et al., 1997; Rodrigo et al., 2007) (Figure 1). When microglia and astrocytes clear Aβ through phagocytosis and intracellular degradation, many transmembrane receptors (e.g., LRP-1 and ATP-binding cassette transporter family) bind to Aβ, and most of these receptors are essential in immune and inflammatory responses (Anthony et al., 2013; Chuang et al., 2016).

### Molecular Receptor Spectrum of Neuroinflammation

The theoretical framework regarding how inflammation interacts with the development of AD has been widened and explored on an unprecedented scale. Internationally renowned pharmaceutical companies have already started multiple clinical drug trials on the anti-inflammatory treatment of AD (Cuello, 2017). Despite cellular components (e.g., microglia, astrocytes, and endothelial cells), an increasing number of receptors, membrane proteins and metabolic enzymes have become candidates for further research and clinical trials. For example, positive modulation of a7 nicotinic acetylcholine receptors (a7nAChRs) had therapeutic potential for its anti-inflammatory effects (Echeverria et al., 2016). The lower dose of colony-stimulating factor 1 receptor (CSF1R) inhibitors led to increased hippocampal-dependent spatial memory (Spangenberg and Green, 2017). Angiotensin II receptor blockers (ARBs) were considered a neuroprotective candidate against early damage factors to neurons, astrocytes, microglia, cerebrovascular endothelial, and BBB; consequently, ARBs prevent cognitive loss and dementia (Saavedra, 2016). Mimetics of glucagon-like peptide 1 (GLP-1) receptor agonists, first designed to treat diabetes, were capable of reducing inflammation, oxidative stress, and apoptotic signaling and protecting memory formation (Hölscher, 2018). In addition to membrane receptors, a substantial number of studies revealed that nuclear receptors could be potential interventional targets to suspend dementia progression in AD (Moutinho and Landreth, 2017). Notably, numerous studies have focused on various inflammatory receptors and diverse signaling pathways. Among these components, chemokine receptors, which are mainly studied in immunology, deserve further attention. The birth of Chinese gene edited infants has greatly enriched scientific theories because some studies showed that CCR5 deficiency could improve both prognosis and neuronal plasticity after stroke and traumatic injury, which encouraged an unprecedented CCR5 research boom (Joy et al., 2019).

## CCR5

### CCR5: Structure, Distribution, and Function

CCR5 belongs to the seven transmembrane G-protein coupled receptors (GPCRs) that transmit signals via heterotrimeric G proteins (Pierce Kristen et al., 2002). CCR5 consists of 7 hydrophobic transmembrane domains with an extracellular N-terminus and cytoplasmic C-terminus, ranging in length from 340 to 370 amino acids (Oppermann, 2004). CCR5 is the receptor for nearly 10 chemotactic proteins of the β chemokine family, which are also named CC chemokines (Griffith et al., 2014). In humans, the gene encoding the CCR5 protein is situated in the short (p) arm at position 21 on chromosome 3.

In the immune system, CCR5 widely resides on the surface of antigen-presenting cells (e.g., macrophages, dendritic cells), effector lymphocytes and regulatory T cells (Griffith et al., 2014). The corresponding ligands are presented on effector T cells and natural killer cells (Nieto et al., 1998; Luther and Cyster, 2001; Hogaboam and Kunkel, 2002). CCR5 regulates chemotaxis and cell activation through interactions with MIP-1α (CCL3), MIP-1β (CCL4), RANTES (CCL5), MCP-2 (CCL8), CCL11 (eotaxin), HCC-1 (CCL14a), and HCC-4 (CCL16) (Alexander et al., 2007).

CCR5 is widely known because it provides access for HIV-1 to invade host immune cells. The gp120 envelope protein is a chemokine mimic; thus, it can bind to CCR5 during HIV infection (Murphy, 2001). Additionally, CCR5 can accelerate the transmission of the R5-strain of the HIV-1 virus (Lieberman-Blum et al., 2008). CCRΔ32 is a 32-base-pair deletion that inserts a premature stop codon into the CCR5 receptor locus, leading to receptor dysfunction. Homozygous carriers of this mutation cannot be infected with M-tropics strains of HIV-1 (De and Stumpf, 2004; Gero et al., 2009; Rieger et al., 2010; Kay and Walker, 2014).

Several lines of cancer cells (e.g., breast and prostate cancer cells), instead of normal epithelial cells, express a high level of CCR5, which help the cancer transformation process (Velasco-Velázquez et al., 2012; Daniela et al., 2014). Furthermore, the chemotherapy resistance of cancer stem cells could be partly accredited to the expression of CCR5 (Jiao et al., 2018).

In the CNS, these receptors are produced by microglia, astrocytes, and endothelial cells (normally undetectable on neurons) (Shukaliak and Dorovini-Zis, 2000; Mi et al., 2009; Subileau et al., 2009). All existing evidence suggests that CCR5 participates in neuroinflammation and neuroimmunology, including microglial activation (Cowell et al., 2006; Bokhari et al., 2009), microglial chemotaxis (Babcock Alicia et al., 2003; Kyung et al., 2010), monocyte/macrophage chemotaxis (Glass William and Lane Thomas, 2003), lymphocyte chemotaxis (Elodie et al., 2003; Glass William and Lane Thomas, 2003), brain development and cell differentiation (Bakhiet et al., 2001; Khan et al., 2003; Mi et al., 2009; Kyung et al., 2010), neuronal transmission (Adler et al., 2005; Chen et al., 2007; Veronica et al., 2008) and anti-microorganism functions (Mishra Saroj and Lothar, 2009; Haworth et al., 2017; Brelot and Chakrabarti, 2018). Under the same conditions, neuronal death was more notable in the brains of CCR5−/− mice than in those of CCR5+/+ mice (Hee et al., 2009). Knockout of the CCR5 gene was associated with the inadequate development and maturation of dopaminergic neurons (Choi et al., 2013).

### CCR5: Signal Pathway

CCR5 activates the cellular signaling pathway through G proteins, pertussis-sensitive heterotrimeric G proteins and G protein-independent pathways (Oppermann, 2004). The binding of a chemokine ligand to CCR5 results in conformational changes in G proteins, which will enable various signaling cascades, including those of the phosphoinositide-3 kinase (PI3K), protein kinase C (PKC), and mitogen-activated protein kinases (MAPK), as well as calcium influx (Oppermann, 2004; Sudarshan et al., 2013). At present, G-protein-independent signaling pathways involving the Janus kinases (JAK), pyk2 kinase, and arrestin pathways have been identified (Corno et al., 2001; Mueller and Strange, 2004). These cascades activate various cellular functions, cytoskeleton recombination, and chemotaxis. Calcium flux is an essential procedure for sequent signal activation in the CNS. Given that various types of cells express CCR5, it is not impossible that its diverse biological effects require more than G-protein-initiated pathways. The different chemokine ligands that CCR5 binds to determine distinct signaling pathways accounting for the pleiotropic effects of chemokine signaling (Mueller et al., 2006; Jenny et al., 2015).

Additionally, blockage of CCR5 signaling could not only lead to rejection of HIV but also elicit neuroprotective mechanisms and promote neuron survival, which will alleviate HIV-associated dementia (Kaul and Lipton, 2006; Christoph and Pett Sarah, 2012; Martin-Blondel et al., 2016).

### The Efficacy of CCR5 Antagonists

The promising development of CCR5 receptor inhibitors is largely based on CCR5’s identity as a necessary pathway for AIDS infection. The only first-generation CCR5 antagonist that the Food and Drug Administration (FDA) approved, Maraviroc, was well tolerated and showed excellent repression of viral load in patients whose highly active antiretroviral therapy (HAART) failed but not in treatment-naïve patients(Lieberman-Blum et al., 2008; Sierra-Madero et al., 2010; Van Lelyveld et al., 2012). The high efficacy of maraviroc has set the tone for second-generation CCR5 antagonists, which account for the failures of aplaviroc and vicriviroc in clinical trials (Gulick et al., 2007; Leeson and Springthorpe, 2007; Stupple et al., 2011). Various modified and innovative second-generation CCR5 antagonists [e.g., piperidine amide compounds (Imamura et al., 2006; Ernst et al., 2008; Stupple et al., 2011), piperazines and diketopiperazines (Liu et al., 2008)] demonstrated guaranteed efficacy in I/II clinical trials, especially the dual CCR5/CCR2 antagonists (Meyer et al., 2013).

However, more data have revealed that CCR5 antagonists function in other disease courses. Dual CCR5/CCR2 antagonists and cenicriviroc could inhibit the progression of non-alcoholic fatty liver disease (Tacke, 2018; Ogawa et al., 2019). In addition, cenicriviroc could reduce liver injury in cholestatic rodents (Yu et al., 2018). Moreover, the inhibition of lymphocyte migration caused by CCR5 blockade could alleviate graft-versus-host disease (Reshef et al., 2019). Could the CCR5 blockade provide relief from AD?

## Ad and CCR5

The definite relationship between AD and CCR5 remains an open debate. Disputable results from basic experimental studies have diverged into two opposed groups. Most of studies demonstrated that the expression of CCR5 contributes to the development of AD (Table 1), while the minority demonstrated that CCR5 could improve memory function in AD (Table 2). However, all existing epidemiologic studies confirmed that there was no association between the CCR5Δ32 allele and AD risk. Therefore, the entanglement of AD and CCR5 urgently needs to be addressed (Table 3).

**TABLE 1:** Studies that CCR5 expression exacerbated AD.

	**Discovery**	**Title**	**Author**	**Model**	**Methods**
**Direct evidence**	Microglia with CCR5 expression are associated with deposition of Aβ.	Immunohistochemical study of the β-chemokine receptors CCR3 and CCR5 and their ligands in normal and Alzheimer’s disease brains.	Xia et al., 1998	Human	Immunohistochemistry of brain tissue of post mortem human being.
	CCR5−/− mice had less activation of microglia and astrocytes after injection of Aβ into lateral ventricle.	Role of the macrophage inflammatory protein- 1alpha/CC chemokine receptor 5 signaling pathway in the neuroinflammatory response and cognitive deficits induced by beta-amyloid peptide.	Passos et al., 2009	Mice	Knockout of mice CCR5 gene.
	CCR5 antagonists attenuated the neuroinflammation of sub cutaneous administration of lipopolysaccharide by decreasing the number of activated microglia and astrocytes.	Morphine induces the release of CCL5 from astrocytes: potential neuroprotective mechanism against the HIV protein gp120.	Avdoshina et al., 2010	Rats	Administration of CCR5 antagonist to rats preinjected with lipopolysaccharide.
	CCR5 participated in the impairment of learning and memory in AD by activating microglia and promoting T cells transendothelial migration.	Peripheral T cells overexpress MIP-1α to enhance its transendothelial migration in Alzheimer’s disease.	Man et al., 2007	Human and rats	CCR5 detection of peripheral blood mononuclear cells from AD patients and healthy controls; Peripheral intravenous injection of Aβ in rats, followed by administration of CCR5 CCR5 antagonist (2D7 mAb).
	The ligands of CCR5, CCL3 and CCL4, were upregulated in microglia isolated from AD patients’ brain and stimulated with Aβ.	Gene expression profiling of amyloid beta peptide-stimulated human post-mortem brain microglia.	Walker et al., 2001	Human	Gene array technology.
	The CCR5 expression of PBMC from AD patients was significantly higher and *in vitro* PBMC culture with Aβ increased the CCR5 expression.	Peripheral chemokine receptors, their ligands, cytokines and Alzheimer’s disease.	Reale et al., 2008	Human	CCR5 detection of PBMC from AD patients and healthy controls.
	The proportion of cells expressing CCR5 (Th1 cells and dendritic cells) was greater in AD patients.	Enhanced Chemokine Receptor Expression on Leukocytes of Patients with Alzheimer’s Disease.	Goldeck et al., 2013	Human	CCR5 detection of PBMC from AD patients and healthy controls.
	Aβ could increase CCR5 expression through cellular signaling of c-Raf, ERK-1/ERK-2, and c-Jun NH2-terminal kinase in PBMC.	Mechanism of amyloid peptide induced CCR5 expression in monocytes and its inhibition by siRNA for Egr-1.	Giri et al., 2005	Human	CCR5 detection of PBMC from AD patients and healthy controls and *in vitro* administration of siRNA for inhibiting CCR5 relevant signal pathways.
	The curcumin inhibited Aβ associated expression of CCR5 by preventing Egr-1 DNA binding to the promoter of CCR5.	Curcumin, the active constituent of turmeric, inhibits amyloid peptide-induced cytochemokine gene expression and CCR5-mediated chemotaxis of THP-1 monocytes by modulating early growth response1 transcription factor.	Giri et al., 2005	Human	Administration of curcumin into PBMC culture *in vitro*.
	The CCR5 antagonist (DAPTA) of monocyte chemotaxis, was proved to reduce chronic neuroinflammation of AD.	Update on D-Ala-Peptide T-Amide (DAPTA): A Viral Entry Inhibitor that Blocks CCR5 Chemokine Receptors; Chemokine receptor 5 antagonist D-Ala-peptide T-amide reduces microglia and astrocyte activation within the hippocampus in a neuroinflammatory rat model of Alzheimer’s disease.	Rosi et al., 2005	Rats	Administration of DAPTA to AD rats.
**Indirect evidence**	Both knockout of CCR5 gene and administration of maraviroc helped new formation of neuronal connections.	CCR5 Is a Therapeutic Target for Recovery after Stroke and Traumatic Brain Injury.	Joy et al., 2019	Mice	Knockout of CCR5 gene and administration of maraviroc.
	Weakening the function of CCR5 in mouse led to enhanced LTP and hippocampus-dependent memory.	CCR5 is a suppressor for cortical plasticity and hippocampal learning and memory.	Cai et al., 2016	Mice	Knockout of CCR5 gene.

**TABLE 2:** Studies that CCR5 expression improved AD.

**Discovery**	**Title**	**Author**	**Model**	**Methods**
CCR5−/− mice showed higher Aβ deposit and impaired long-term and spatial memory.	CCR5 deficiency induces astrocyte activation, Abeta deposit and impaired memory function.	Yong et al., 2009	Mice	Knockout of mice CCR5 gene.
CCR5 expression reduced in amyloid precursor protein plus presenilin-1 (APP/PS1) mice.	Changes in Chemokines and Chemokine Receptors Expression in a Mouse Model of Alzheimer’s Disease.	Obrador et al., 2019	Mice	Detection of CCR5 in APP/PS1 mice by quantitative RT-PCR and Western-blot techniques.
CCR5 reduction resulted in an increase of Aβ deposits and impairment of memory.	CCR5 deficiency accelerates lipopolysaccharide-induced astrogliosis, amyloid-beta deposit and impaired memory function.	Hwang et al., 2016	Mice	Knockout of CCR5 gene in mice.
CCR5 gene expression was significantly reduced over time in Tau-P201L mice.	CXCR4 involvement in neurodegenerative diseases.	Andreassen et al., 2018	Mice	Tau transgenic mouse models.
CCR5−/− mice showed higher Aβdeposition.	CCR5 deficiency induces astrocyte activation, Aβ deposit and impaired memory function.	Yong et al., 2009	Mice	Knockout of CCR5 gene in mice.

**TABLE 3:** Studies that show no association between CCR5 expression and AD development.

**Discovery**	**Title**	**Author**	**Model**	**Methods**
The CCR5Δ32 gene mutation in Spanish is not associated with the AD risk.	The chemokine receptor CCR5-Delta32 gene mutation is not protective against Alzheimer’s disease.	Onofre et al., 2004	Human	Epidemiologic study.
The CCR5Δ32 allele did not did not contribute to the risk of AD.	Chemokines (RANTES and MCP-1) and chemokine- receptors (CCR2 and CCR5) gene polymorphisms in Alzheimer’s and Parkinson’s disease.	Huerta et al., 2004	Human	Epidemiologic study.
No significant difference was shown in the distribution of CCR5 between AD patients and healthy controls in Iran.	Ccr2-64i and Ccr5 Δ32 Polymorphisms in Patients with Late-Onset Alzheimer’s disease; A Study from Iran (Ccr2-64i And Ccr5 Δ32 Polymorphisms in Alzheimer’s disease).	Khorshid et al., 2012	Human	Epidemiologic study.
No significant differences was demonstrated in genotype distribution and CCR5Δ32 allelic frequency both in women and in men in Italy.	Association between the Polymorphism of CCR5 and Alzheimer’s Disease: Results of a Study Performed on Male and Female Patients from Northern Italy.	Balistreri et al., 2006	Human	Epidemiologic study.

### The Major Group: CCR5 Exacerbates AD (Table 1)

**FIGURE 2:**
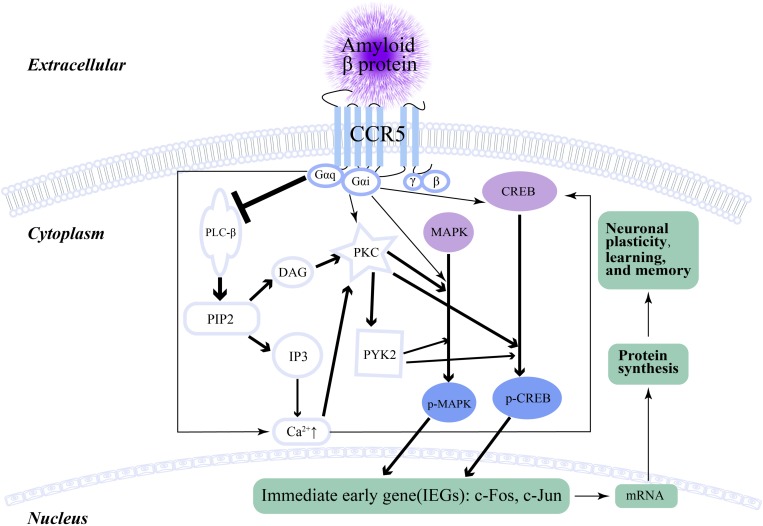
The signaling pathway of CCR5 and neuronal plasticity. The arrow represents promotion, while the ⊥ represents inhibition. The thickness of the arrow and ⊥ indicates the intensity of promotion or inhibition.

Memory, learning and plasticity processes in hippocampal and cortical circuits involve electrochemical activity of neurons, including long-term potentiation (LTP), release of glutamate, activation of *N*-Methyl-D-aspartic acid (NMDA) receptors and α-amino-3-hydroxy-5-methyl-4-isoxazolepropionic acid (AMPA) receptors, and the generation of new dendritic spines and axons. Cai et al. (2016) reported that weakening the function of CCR5 in the mouse barrel cortex improved MAPK/CREB signaling, which resulted in enhanced spike-timing-dependent plasticity and experience-dependent plasticity (Figure 2). Consequently, LTP and hippocampus-dependent memory significantly improved, while neuronal CCR5 overaction led to memory deficits (Cai et al., 2016). This cornerstone study underlies the foundation of the relationship between memory and CCR5, which also implies a potential discoverable link between CCR5 and AD. The increase in both CCR5 and CCR3 on some reactive microglia was found in AD patients and associated with amyloid deposition (Xia et al., 1998). After the administration of Aβ into the lateral ventricle of CCR5−/− mice, the activation of microglia and astrocytes was decreased compared with that in CCR5 wild-type mice (Passos et al., 2009). CCR5−/− mice showed decreased astrocytosis and microgliosis in the hippocampus after Aβ injection, which resulted from decreased expression of cyclooxygenase-2 and inducible nitric oxide (NO) synthase, as well as reduced activation of nuclear factor-κB (NF-κB), activator protein-1 and cyclic AMP response element-binding protein (Passos et al., 2009). In humans, there were more widespread reactive astrocytes with CCR5 expression in AD than in healthy (Xia et al., 1998) controls. In addition, the impairment of memory and synaptic dysfunction caused by Aβ were alleviated in CCR5−/− mice (Passos et al., 2009). Similarly, both knockout of the CCR5 gene and administration of maraviroc (FDA-approved anti-HIV drug) could heighten plasticity in the premotor cortex proximal to the stroke site and upregulate CREB and DLK signaling in neurons, which finally aided in the formation of new connections in the contralateral premotor cortex (Joy et al., 2019). Although this conspicuous study did not connect CCR5 with AD, it suggested that CCR5 and its signaling pathways had a pivotal impact on the generation and regeneration of dendritic spines and synapses. Peripheral blood immune cells can filter into the CNS with the help of CCR5 (Man et al., 2007). Man et al. (2007) suggested that CCR5 participated in the impairment of learning and memory in AD by activating microglia and promoting T cell transendothelial migration through the Rho/ROCH pathway. CCL3 and CCL4, as ligands for CCR5, were upregulated in microglia isolated from AD patient brains and stimulated with Aβ (Walker et al., 2001). In addition, peripheral blood mononuclear cell (PBMC) analysis suggested that the expression of both CCR5 and CCR2 were augmented in AD patients compared with those in control subjects (Reale et al., 2008). Further experiments suggested that acetylcholinesterase inhibitor (ACEI) was capable of decreasing the expression of CCR5 and CCR2 in the PBMCs of AD patients, while *in vitro* administration of Aβ increased their expression in PBMCs (Reale et al., 2008). Similar results were also obtained by Goldeck et al. (2013), who found that the distribution of cells expressing CCR4 (expressed on Th2 cells) and CCR5 (Th1 cells and dendritic cells) was also greater in patients and was more distinguished on CD4+ than CD8+ T cells. A CCR5 antagonist could reduce the number of active astrocytes and microglia after lipopolysaccharide injection, which implied that CCR5 was involved in neuroinflammation related to AD (Combarros et al., 2004). Giri et al. (2005) indicated that Aβ could increase CCR5 expression through cellular signaling of c-Raf, ERK-1/ERK-2, and c-Jun NH2-terminal kinase in PBMCs. As mentioned above, CCR5 is involved in neuroinflammation, wherein various CCR5 antagonists can attenuate the neuroinflammation associated with AD. D-Ala-peptide T-amide (DAPTA), a kind of CCR5 antagonist of monocyte chemotaxis, was shown to reduce chronic neuroinflammation by blocking the release of the proinflammatory cytokines TNF-α and IL-1 (Ruff et al., 2003; Rosi et al., 2005). After the administration of Egr-1 siRNA, Aβ-associated CCR5 expression and its concomitant ligands decreased significantly (Giri et al., 2005). Curcumin also inhibited the Aβ-associated expression of CCR5 by preventing Egr-1 DNA binding to the promoter of CCR5 (Giri et al., 2010).

### The Minor Group: CCR5 Improves AD (Table 2)

However, CCR5 expression was reduced, and both chemokine CCL3 and CCL4 chemokine expression levels were augmented in amyloid precursor protein plus presenilin-1 (APP/PS1) mice, which are a common model for AD studies (Obrador et al., 2019). Additionally, Hwang et al. (2016) showed that CCR5 reduction led to higher Aβ deposition and impairment of memory. Lipopolysaccharide injection of CCR5 knockout mice significantly increased astrogliosis and Aβ deposition compared with those in CCR5 wild-type mice (Hwang et al., 2016). Within the hippocampus and cerebral cortex of Tau-P201L mice, which is another frequently used model for AD, CCR5 gene expression was significantly reduced over time compared with that in wild-type mice, while no distinction was found between other brain regions(Andreassen et al., 2018). CCR5−/− mice showed a higher level of Aβ, which was related to astrocyte activation and CCR2 overexpression. These changes finally caused memory impairments (Yong et al., 2009).

### No Epidemiological Link Between AD and CCR5 (Table 3)

Despite the fact that experimental studies were full of controversies and the conclusive relationship between AD and CCR5 remained unknown, the epidemiological evidence was certain and negative. Several epidemiological studies confirmed that there was no differential distribution of the CCRΔ32 deletion in AD patients and controls in Italy (Balistreri et al., 2006). Moreover, no differences were observed by gender stratification, by the presence of the ApoE q4 allele, or by the age at onset in genotype distribution and allelic frequency (Balistreri et al., 2006). Identical results were found in Spanish and Iran: the CCR5Δ32 allele was not a correlative factor for AD (Galimberti et al., 2004; Onofre et al., 2004; Salvador et al., 2004).

## Conclusion and Outlook

The definite mechanism of AD remains unclear, but our understanding of AD has far exceeded the two typical pathological manifestations—Aβ and tau. The neuroinflammation theory of AD is fascinating an increasing number of researchers. Currently, more than 5000 studies about AD and neuroinflammation are available on PubMed, and the number is expanding. CCR5 expression was strongly related to microglia and inflammation, which validated an inseparable relationship between inflammation, Alzheimer’s disease, and CCR5 (Andreassen et al., 2018). As the most notable receptor among the chemokine receptor family, the accurate function and subsequent signaling pathways of CCR5 have been well studied. Available epidemiological evidence could provide no connection between the CCR5Δ32 gene and AD (Onofre et al., 2004; Salvador et al., 2004; Balistreri et al., 2006). However, given that all of the epidemiological evidence was based on genotype and performed more than a decade ago, potential links may lie deeper than the superficial genotype, such as translational modulation, proteomics, and epigenetics. Despite the controversy, it cannot be denied that CCR5 plays an important role in the process of LTP, cortical plasticity, learning and memory (Cai et al., 2016). We posited that the reason for experimental conflicts between CCR5 and AD are as follows: the animal models and the methods the authors used for acquiring AD models were varied. Furthermore, compared with administration of small interfering RNA (siRNA), maraviroc and other antagonists, the effect of total knockout of the CCR5 gene could lead to an enormous impact on cellular signal pathways inside the membrane. Further investigation should take these factors into account. Future studies of molecular mechanisms of subsequent intracellular signal pathways caused by CCR5 activation would shed new light on this entanglement between AD and CCR5.

## Author Contributions

TL contributed significantly on this review. JZ reviewed and proposed valuable modifications.

## Conflict of Interest Statement

The authors declare that the research was conducted in the absence of any commercial or financial relationships that could be construed as a potential conflict of interest.
